# Genome Sequence of Rickettsia bellii Illuminates the Role of Amoebae in Gene Exchanges between Intracellular Pathogens

**DOI:** 10.1371/journal.pgen.0020076

**Published:** 2006-05-12

**Authors:** Hiroyuki Ogata, Bernard La Scola, Stéphane Audic, Patricia Renesto, Guillaume Blanc, Catherine Robert, Pierre-Edouard Fournier, Jean-Michel Claverie, Didier Raoult

**Affiliations:** 1 Structural and Genomic Information Laboratory, Centre National de la Recherche Scientifique UPR-2589, Institut de Biologie Structurale et Microbiologie, Parc Scientifique de Luminy, Marseille, France; 2 Unité des Rickettsies, Centre National de la Recherche Scientifique UMR-6020, IFR-48, Faculté de Médecine, Université de la Méditerranée, Marseille, France; The Institute for Genomic Research, United States of America

## Abstract

The recently sequenced Rickettsia felis genome revealed an unexpected plasmid carrying several genes usually associated with DNA transfer, suggesting that ancestral rickettsiae might have been endowed with a conjugation apparatus. Here we present the genome sequence of *Rickettsia bellii,* the earliest diverging species of known rickettsiae. The 1,552,076 base pair–long chromosome does not exhibit the colinearity observed between other rickettsia genomes, and encodes a complete set of putative conjugal DNA transfer genes most similar to homologues found in Protochlamydia amoebophila UWE25, an obligate symbiont of amoebae. The genome exhibits many other genes highly similar to homologues in intracellular bacteria of amoebae. We sought and observed sex pili-like cell surface appendages for *R. bellii.* We also found that R. bellii very efficiently multiplies in the nucleus of eukaryotic cells and survives in the phagocytic amoeba, *Acanthamoeba polyphaga.* These results suggest that amoeba-like ancestral protozoa could have served as a genetic “melting pot” where the ancestors of rickettsiae and other bacteria promiscuously exchanged genes, eventually leading to their adaptation to the intracellular lifestyle within eukaryotic cells.

## Synopsis

Obligate intracellular pathogens, by their isolated way of life, are thought to have little opportunity to exchange DNA with other microbes, making their genome relatively immune to horizontal gene transfer. Rickettsia bellii is the earliest diverging species of *Rickettsia,* a group of obligate intracellular bacteria causing human diseases such as epidemic typhus. Sequencing and computer analysis of its genome now provides evidence that many genes were transferred between the ancestors of *Rickettsia* and other microbes living inside amoebae, including the human pathogen Legionella pneumophila and the chlamydia-related symbiont *Protochlamydia amoebophila.* Remarkably, R. bellii exhibits a complete set of predicted genes for conjugative DNA transfer. Microscopic analyses confirmed this prediction by revealing bacterial mating through sex pili-like surface appendages. Furthermore, the authors demonstrate that R. bellii can survive in an amoeba for a long period of time. Taken together, these results suggest that amoeba-like ancestral protozoa acted as a “breeding ground” for different bacteria, promoting acquisition of the gene repertoire required to infect the cells of higher eukaryotes.

## Introduction

**Figure 1:**
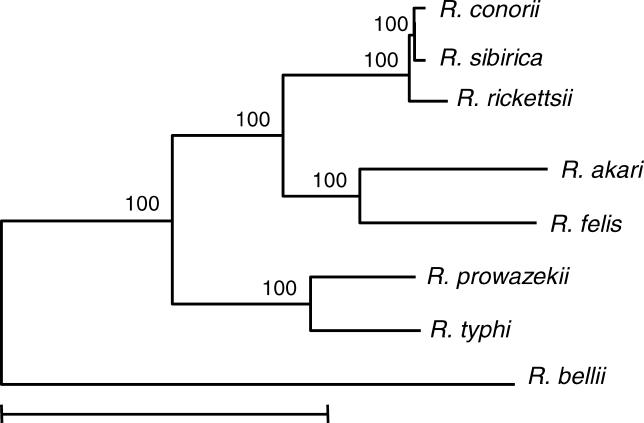
Phylogenetic Relationships of R. bellii with Other Rickettsiae The tree was built using the comparison of concatenated nucleotide sequences of the 16S rDNA, *gltA, pheS, pheT, nusA, valS,* and *smpB* genes using the neighbor-joining method with Kimura-2 parameter. Bootstrap values are indicated at the nodes. The tree was rooted using the orthologous sequences of Anaplasma marginale as an outgroup (not shown). The scale bar corresponds to 5% divergence in nucleotide sequences.

Rickettsiae are arthropod associated obligate intracellular Gram-negative bacteria causing mild to severe diseases in humans. They are classified into three groups based on phylogenetic analyses [1,2]. The typhus group (TG) includes the louse-borne R. prowazekii (the agent of epidemic typhus) and the flea-borne R. typhi (the agent of endemic typhus). The spotted fever group (SFG) includes tick-borne R. rickettsii (the agent of Rocky Mountain spotted fever), R. conorii (the agent of Mediterranean spotted fever), R. africae (the agent of African tick bite fever), R. sibirica (the agent of North Asian tick-borne fever), mite-borne R. akari (the agent of rickettsialpox), and flea-borne R. felis (the agent of flea-borne spotted fever). R. bellii is the only known species representing a third group that diverged prior to the separation of the other two groups (Figure 1). R. bellii is the most common rickettsia found in ticks in America, in which it is transovarially transmitted. It has been found in various hard ticks, including species of *Dermacentor* and *Amblyomma.* It is also the sole rickettsia found in both soft and hard ticks, therefore exhibiting the largest arthropod host range among known rickettsiae (Table S1). The recently sequenced genome of R. felis revealed an unexpected plasmid carrying several genes usually associated with DNA transfer [3]. This suggested the existence of a fully operational conjugation system in ancestral rickettsiae. The R. bellii genome was estimated to be about 1.6 Mb long [4]. Given that its genome size is comparable to R. felis and larger than other *Rickettsia,* together with its early branching phylogenetic position, we reasoned that R. bellii might have retained ancestral features lost in other lineages, and that its genome sequence might help reconstruct the early stage of rickettsia evolution. Here we present the sequence of the R. bellii genome, which suggests that numerous gene exchanges took place between ancestral rickettsiae and amoeba-associated bacteria probably mediated by conjugation within co-infected amoebae. In support of this hypothesis, we show that R. bellii exhibits sexual pili and is able to survive in amoebae. In addition, we present preliminary results indicating that R. bellii is pathogenic to mammals.

## Results

### General Features of the Genome

The genome of R. bellii consists of a single circular chromosome of 1,522,076 base pairs (bp) with a G + C content of 31.7% (Figure S1). This chromosome is the largest among those currently sequenced for species of the order *Rickettsiales.* We identified 1,429 protein-coding genes (open reading frames [ORFs]), 34 tRNA genes, one set of genes for rRNA, and three other structural RNA genes (Table 1). Much of the genome (85.2%) corresponds to coding regions. Of the 1,429 ORFs, 985 (69%) were annotated with putative functions. Using RepeatFinder, we identified 217 repeated DNA sequences (≥50 bp) encompassing 3.7% of the genome. Among other sequenced rickettsiae, only R. felis exhibits a comparable level of repeat content (4.3%) [3]. We also identified 526 *Rickettsia* palindromic elements [5,6], of which 53 are in protein-coding genes and one is inserted in the tmRNA-coding gene. R. bellii possesses 178 putative protein-coding genes lacking homologues detectable by BLAST (E-value < 0.001) in the previously sequenced *Rickettsia* species (i.e., *R. felis, R. conorii, R. typhi,* and R. prowazekii), and 146 genes that exhibit homologues but no orthologues (defined by reciprocal BLAST best hits) in these *Rickettsia*. All together, 324 R. bellii specific genes lack clear orthologues in other *Rickettsia.*

**Table 1:**
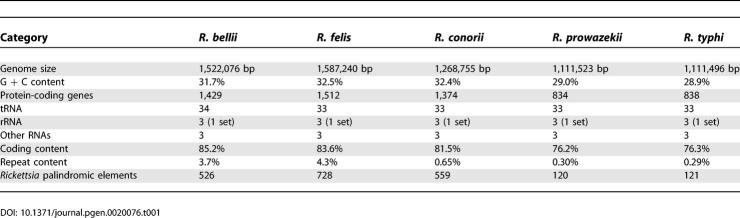
R. bellii Genome Features

### Genome Structure

**Figure 2:**
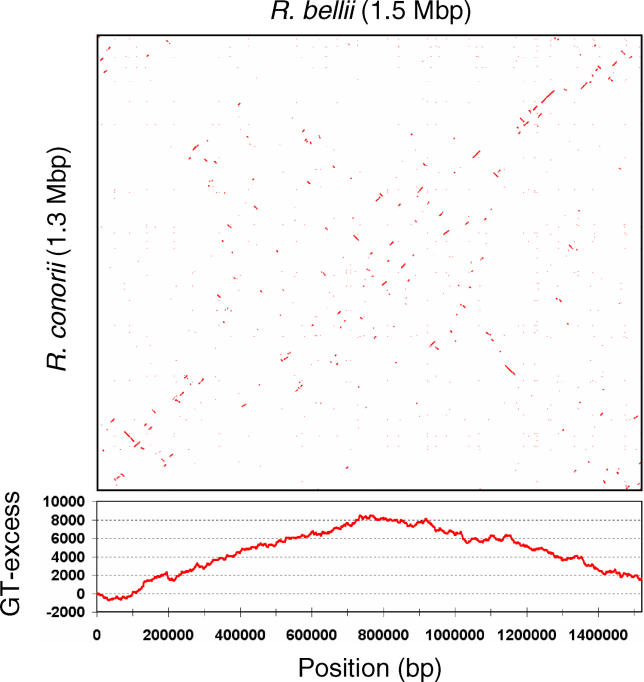
Comparison of R. bellii and R. conorii Genomes, and the Cumulative Nucleotide-Excess Curve for the R. bellii Genome In the top panel, dots represent homologous genomic segments detected by BLASTN (E-value < 0.001). Bottom panel shows the GT-excess plot.

Previously sequenced *Rickettsia* genomes exhibited a high level of colinearity with each other. The R. bellii genome is the first to exhibit little colinearity with other *Rickettsia* genomes, suggesting that it has been shuffled in the course of evolution (Figure 2). The longest R. bellii genomic segment colinear with the genome of R. conorii encompasses only 34 ORFs (from RBE_1152 to RBE_1185). Repeated DNA sequences including transposases were identified at the extremities of some of the R. bellii genome segments that are colinear with the other rickettsia genomes (unpublished data). This suggests the involvement of these repeats in R. bellii genome shuffling. A dot-plot comparison between R. bellii and R. conorii genomes exhibits an X-shaped pattern (Figure 2), indicating that chromosomal inversions symmetrical with respect to the origin-terminus axis dominated the genome rearrangement events [7,8]. It is known that such inversions tend to maintain the nucleotide/gene frequency skew between leading and lagging strands. Consistently, the genome exhibits a smooth cumulative nucleotide skew (GT-excess) [9] (Figure 2).

### R. bellii–Specific Genes

Remarkably, 324 R. bellii–specific genes include 14 genes that are probably responsible for conjugative DNA transfer (see below). The 178 genes lacking homologues in other *Rickettsia* include 108 genes of unknown functions, 40 ORFs encoding transposases, 2 genes encoding type I restriction-modification system proteins, 1 phage-related protein gene, ten genes involved in conjugative transfer, and 15 genes with putative functions newly identified in rickettsia genomes (Table S2). The 146 genes lacking orthologues in other rickettsiae include nine ORFs encoding transposases, four genes involved in type I restriction-modification systems, one integrase gene, and one phage-related protein gene. Overall, the R. bellii–specific genes are enriched in those associated to genome plasticity (e.g., transposases, integrases, or phage-related proteins).

*Rickettsia* genomes are known to harbor many split genes (i.e., fragmented into several pieces of ORFs by internal stop codons), a signature of progressive genome degradation processes. As previously defined [10], a split gene corresponds to two or more consecutive ORFs that are aligned collinearly with longer homologues. We found that several transposase genes of R. bellii are split into two ORFs. For the purpose of similarity analysis, we computationally concatenated those split genes, and obtained 39 transposase sequences. These transposases were classified into four families (family-1 to family-4; Figure S2) using a single linkage clustering approach (i.e., a transposase protein was attributed to a family if its BLASTP alignment with at least one member of the family had an E-value < 10^−5^). The family-1 comprises 27 members, including 13 split genes. The family-2 comprises nine genes, including two split genes and a fragmented gene. The family-3 comprises two genes, including one split gene and a fragmented gene. The family-4 comprises a single gene in *R. bellii.* Other rickettsiae also exhibit several members of family-2, −3, and −4. Members of family-1, −2, and −3 have been found in a wide variety of bacteria. We noticed that some of the split transposase genes exhibit an identical pattern of fragmentation. Due to the fragmentations of ORFs, those split genes may not encode a functional transposase. The identical splitting pattern in those genes may originate in the duplication/transposition of an already split gene through functional polypeptides encoded by full-length transposase genes.

The Sca family is one of the largest protein families in *Rickettsia.* Several members of the family, such as rOmpA [11], rOmpB [12], and Sca4 [13], are known antigenic determinants of SFG or TG rickettsiae. Recently, R. conorii rOmpB was shown to be involved in the adhesion to host cells by interacting with membrane-associated Ku70 [14]. R. bellii exhibits serological cross-reactivities with SFG and TG rickettsiae. In the R. bellii genome, we identified 11 genes that belong to the *sca* family (Table S3): *sca1* (RBE_0170), *sca2–6* (the preduplication ancestral gene of the *R. akari sca2* and *sca6;* RBE_1280), *sca3* (RBE_0761/0762), *sca4* (RBE_0769), *ompB*/*sca5* (RBE_0184), *sca8* (RBE_0355/0354/0353), *sca9* (RBE_0146/0147), *sca13* (RBE_0148/0149), *sca14* (RBE_0384), *sca15* (RBE_0918), and *sca16* (RBE_0270). Among these, *sca14, sca15,* and *sca16* lack orthologues in other rickettsaie, and are thus unique to *R. bellii.*

R. bellii possesses genes for toxin antitoxin (TA) systems (eight for toxin and nine for antitoxin) as well as ten paralogous genes for *spoT,* of which six are specific to *R. bellii.* TA loci (13) were recently identified in the genome of R. felis [3]. R. conorii also exhibits 5 ORFs for toxin and 6 ORFs for antitoxin. TG rickettsiae exhibit no ORFs for TA systems. Genes for TA systems were originally identified in plasmids, but may also be found in the chromosomes of free-living bacteria [15]. TA systems participate in the cascade of the stringent response pathway induced by alarmones (ppGpp and pppGpp) whose intracellular concentration is regulated by SpoT/RelA enzymes. TA systems are rare in most obligate intracellular bacteria, which multiply in constant environments, in which these stress response pathways are likely dispensable [15,16]. The presence of the high number of TA loci in R. bellii and SFG rickettsiae, as well as the ubiquitous presence of multiple copies of *spoT* genes in all sequenced rickettsiae, suggest that the stringent response pathway is still important for those obligate intracellular bacteria. The role of TA systems in the stringent response might have been lost in TG rickettsiae.

Finally, we also identified 25 ORFs encoding ankyrin repeats and nine ORFs encoding tetratricopeptide repeats. Both motifs are associated with protein–protein interactions. The abundance of these sequence motifs is a shared feature of R. bellii and R. felis genomes.

### Chromosome-Encoded *tra* Gene Cluster

**Figure 3:**
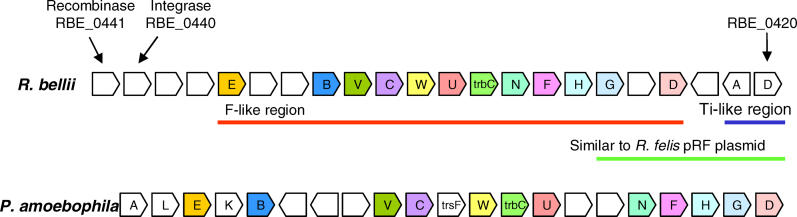
Alignment of the *tra* Gene Clusters Found in the Chromosomes of R. bellii and P. amoebophila Homologous genes are indicated by the same color. For *R. bellii,* regions similar to *tra* genes in E. coli F plasmid, A. tumefacience Ti plasmid, and R. felis pRF plasmid are indicated by red, blue, and green bars, respectively.

The R. bellii chromosome exhibits a *tra* gene cluster (Figure 3), which is predicted to encode a type IV secretion system (T4SS) for conjugal DNA transfer [17]. The *tra* gene cluster is composed of an “F-like region” and a “Ti-like region,” The F-like region exhibits a high level of homology and colinearity with the well-characterized *tra*-region of Escherichia coli F plasmid [18] and with the recently described *tra*-region of the obligate amoeba-endosymbiont Protochlamydia amoebophila UWE25 [19–21]. The Ti-like region is similar to a part of the *tra*-region of Agrobacterium tumefaciens Ti plasmid [22]. The G + C composition of the *tra* gene cluster is 32.7%, similar to that of the entire genome.

The F-like region of the *R. bellii tra* gene cluster encodes at least 12 putative proteins involved in conjugative DNA transfer (Table 2). These correspond to most of the core T4SS proteins (TraE_F_, TraB_F_, TraV_F_, TraC_F_, and TraG_F_ [N-terminal domain]) as well as to all the auxiliary proteins (TraF_F_, TraG_F_ [C-terminal domain], TraH_F_, TraN_F_, TraU_F_, TraW_F_, and TrbC_F_) essential for pilus assembly and mating pair stabilization [17]. The presence of these genes strongly suggests that the *R. bellii tra* gene cluster encodes a functional F-like T4SS system. The Ti-like region exhibits two ORFs homologous to TraA_Ti_ and TraD_Ti_. TraA_Ti_ is thought to carry the nickase and helicase activities for initiating DNA transfer [22]. The function of TraD_Ti_ is unknown. R. bellii does not possess clear homologues of three known T4SS core proteins: TraA_F_ (pilin subunit, 112–128 aa), TraL_F_ (93–105 aa), and TraK_F_ (299–410 aa) [17]. We noticed that the hypothetical ORFs (RBE_0435 [110 aa], RBE_0436 [151 aa], RBE_0438 [102 aa], and RBE_0439 [594 aa]) in the *R. bellii tra* gene cluster are comparable in size to the three missing *tra* genes. We previously found a similar but partial *tra* gene cluster in the R. felis pRF plasmid (Figure 3) [3]. The *R. felis tra* region exhibits only homologues for *traG*_F_, *traD*_F_, *traA*_Ti_, and *traD*_Ti_. *traG*_F_, *traD*_F_, and *traA*_Ti_ are split in R. felis. The newly found *R. bellii tra* gene cluster thus encodes the most complete DNA transfer machinery found so far in *Rickettsia.*

**Table 2:**
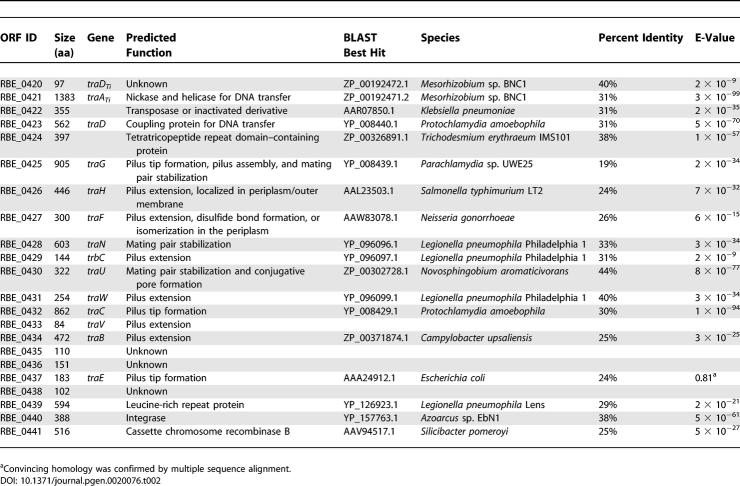
R. bellii ORFs Putatively Involved in Conjugative DNA Transfer

### Sex Pili-Like Surface Appendage of R. bellii

**Figure 4:**
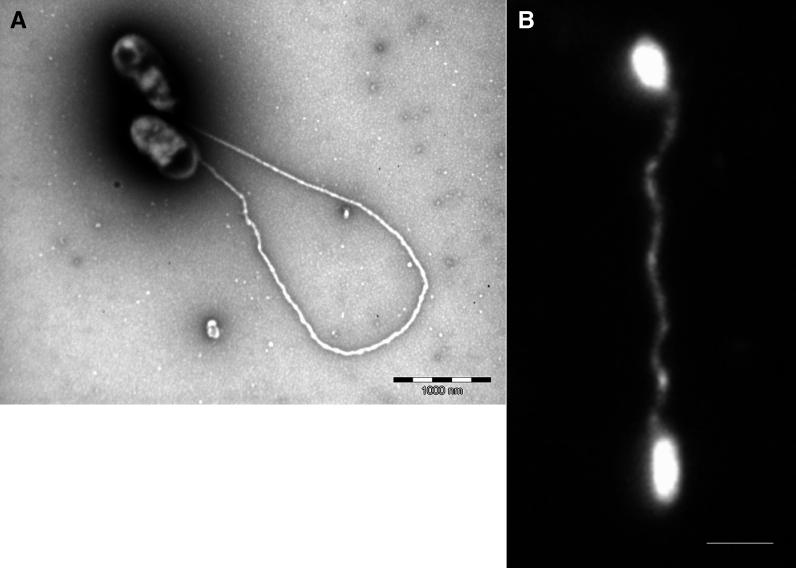
Sex Pili-Like Surface Appendage of R. bellii (A) Electron micrograph of pili observed after negative staining. R. bellii shows long and flexible pilus between 2 bacteria observed from supernatant of XTC-2 cells infected for 48 h at 28 °C. Bacteria were fixed by addition (1:1) of 2% formaldehyde-saline and samples were applied to copper Formvar grids (Electron Microscopy Sciences) for 2 min before negative staining with 1% phosphotungstic acid (pH 6.5) for 2 to 4 min. Samples were examined with a Hitachi 7000 transmission electron microscope. (B) Immunofluorescence performed on the same bacterial culture. R. bellii were probed with a rabbit polyclonal anti-R. belli antibody and with a FITC-conjugated goat anti rabbit Ig as secondary antibody. Slides were examined with an Olympus BX 51 microscope (magnification 100×).

Conjugation through a F-like T4SS system requires a lasting physical contact between the mating pair of bacterial cells. The contact is achieved by pili, filamentous appendages on the bacterial cell surfaces. Transmission electron microscopy revealed such filaments on the surface of R. bellii (Figure 4A). The pili of *R. bellii,* which can also be observed by immunofluorescence staining (Figure 4B), are long and flexible, and physically connect bacteria with each other. In contrast to short and rigid pili that are usually involved in mating on solid surfaces, such long and flexible pili are known to form stable mating pairs in liquid media [23]. These filamentous appendages are likely involved in the early stages of conjugation.

### Phylogenetic Analysis of *tra* Genes

We conducted phylogenetic analyses for every ORF of the F-like region (Figure S3) except TraV, for which a single P. amoebophila homologue was identified by a BLAST search. The analyses pointed out close evolutionary relationships between R. bellii and P. amoebophila sequences for several components of the F-like region. R. bellii and P. amoebophila ORFs formed an exclusive group in four of the 11 reconstructed phylogenetic trees (TraG_F_, TraD_F_, TraB_F_, and TraH_F_). For TraD_F_ and TraH_F_, the grouping is supported by high bootstrap values (i.e., 84% for TraD_F_, 98% for TraH_F_). For the remaining seven ORFs, no significantly supported clade containing R. bellii was obtained, except that the tree for TrbC_F_ indicates an evolutionary relationship between R. bellii TrbC_F_ and its homologues found in Serratia marcescens and Salmonella typhi plasmids (an 81% bootstrap value). We also generated phylogenetic trees for the two ORFs encoded in the Ti-like region. The tree for the TraA_Ti_ supports a phylogenetic affinity of the R. bellii ORF, with homologues found in Legionella pneumophila Paris and *Vibrio vulnificus,* while the phylogeny for TraD_Ti_ is not informative regarding the position of the R. bellii ORF.

**Figure 5:**
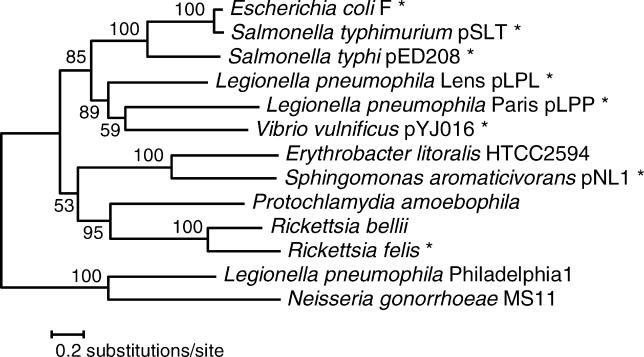
Phylogenetic Tree of the Proteins Encoded in the F-Like *tra* Gene Clusters The tree was built using a maximum likelihood method with JTT substitution model and midpoint rooting based on the concatenated sequence alignment of TraD_F_ and TraG_F_. Plasmid encoded sequences are indicated by asterisks. Other sequences are encoded in the chromosomes.

To further examine the phylogenetic affinity between R. bellii and P. amoebophila suggested for several ORFs of the F-like region, we concatenated the alignments of the six longest ORFs (TraB_F_, TraC_F_, TraD_F_, TraG_F_, TraH_F_, and TraN_F_) and conducted phylogenetic reconstructions. The maximum likelihood tree supports a clustering of R. bellii and P. amoebophila sequences with a 100% bootstrap value (Figure S4). We also generated a maximum likelihood phylogenetic tree including R. felis homologues based on the concatenated sequence alignment of TraD_F_ and TraG_F_, which are the two ORFs encoded in the F-like region of the R. felis plasmid (Figure 5). In this tree, R. bellii clusters with R. felis (a 100% bootstrap value), suggesting a common origin for the chromosome-encoded *tra* gene cluster of R. bellii and the plasmid-encoded *tra* cluster of *R. felis.* The grouping of the rickettsial homologues and *P. amoebophila* homologues was also supported by a high bootstrap value (95%). We applied three additional tree inference approaches (the balanced minimum evolution, the neighbor joining, and the maximum parsimony approaches) for the two concatenated alignments. We obtained significant bootstrap supports for the sister grouping of *Rickettsia* and P. amoebophila by all the approaches for the two datasets, except for the TraD_F_/TraG_F_ tree obtained by the maximum parsimony method (Table S4). Overall, the phylogenetic affinity between *Rickettsia* and P. amoebophila invokes the lateral transfer of at least several *tra* genes between these two distant intracellular bacteria. This does not rule out that the evolutionary history of the entire *tra* gene cluster of R. bellii could have involved a more complex scenario than a single event of direct lateral transfer, as suggested by the incongruent trees obtained for several genes (e.g., TrbC_F_ and TraA_Ti_).

### Gene Exchanges between Intracellular Bacteria of Amoebae

We noticed that many R. bellii ORFs exhibit a high level of sequence similarity to homologues found in two intracellular bacteria of amoebae: *Legionella pneumophila,* a facultative intracellular bacterium of amoebae, and *P. amoebophila.* BLAST homology searches (E-value < 10^−5^) against the SWISS-PROT/TrEMBL database (excluding sequences of Rickettsia spp.) detected 46 and 17 R. bellii ORFs best matching to the sequences of L. pneumophila and *P. amoebophila,* respectively.

**Figure 6:**
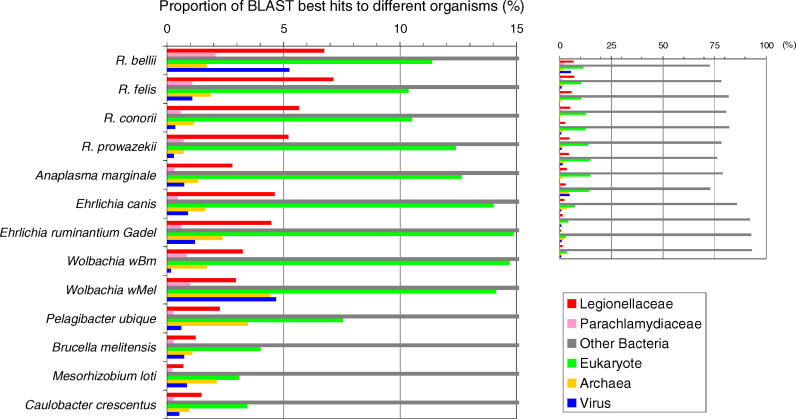
Proportions of BLAST Best Hits to Different Organism Groups The percentages are given relative to the total number of ORFs exhibiting database hits (E-value < 10^−5^).

To assess the significance of the enrichment of *Legionella*-like or *Protochlamydia*-like sequences in *R. bellii,* we performed BLAST searches (E-value < 10^−5^) against the database now excluding all the sequences of Alphaproteobacteria, and counted the number of best hits against *Legionellaceae* or *Parachlamydiaceae.* Among 1,065 ORFs with database hits (E-value < 10^−5^), 94 ORFs (8.8%) exhibited their highest scores to *Legionellaceae* (72 ORFs) or *Parachlamydiaceae* (22 ORFs). We performed the same analyses for other genomes of Alphaproteobacteria (Figure 6). We used eight previously sequenced *Rickettsiales* (obligate intracellular bacteria; 1.1 Mb to 1.6 Mb genomes), and four Alphaproteobacteria outside *Rickettsiales: Pelagibacter ubique* (a free-living marine bacterium with a small genome of 1.3 Mb), Brucella melitensis (a facultative intracellular pathogen of mammals; 3.3 Mb), *Caulobacter crescentus* (a free-living oligotrophic bacterium; 4.0 Mb), and Mesorhizobium loti (a legume symbiont; 7.6 Mb). The latter four species were sampled to cover the wide range of ecological niches and lifestyles found among Alphaproteobacteria. The result (Figure 6) shows that the enrichment of *Legionella*-like or *Protochlamydia*-like sequences of R. bellii (8.8%) is significantly higher than those for the four Alphaproteobacteria outside *Rickettsiales* (0.9% for M. loti to 2.5% for *P. ubique;* Fisher's exact test *p* < 10^−9^). For the eight *Rickettsiales* genomes, the corresponding proportions of *Legionellaceae* or *Parachlamydiaceae* best hits are from 3.1% *(Anaplasma marginale)* to 8.2% *(R. felis),* which are comparable to or slightly lower than that for *R. bellii.*

We hypothesized that the observed abundance R. bellii ORFs similar to homologues of the two groups of intra-amoeba bacteria could be at least partially due to horizontal gene transfers (HGTs) between the ancestor of R. bellii and these amoebal parasites. In order to identify potential candidate genes for such HGTs, we prepared a database by removing sequences of *Rickettsiales* from the SWISS-PROT/TrEMBL database and performed bidirectional BLAST searches (E-value < 10^−5^). We first carried out BLAST searches for R. bellii ORFs against this database (without *Rickettsiales* sequences), and extracted the sequences of *Legionellaceae* that got best matches from R. bellii ORFs. Next, each of the *Legionellaceae* sequences was searched against the same database plus all the R. bellii ORFs. When the *Legionellaceae* sequence exhibited its best BLAST score (omitting hits against *Legionellaceae*) to the R. bellii ORF that detected the *Legionellaceae* sequence in the first BLAST search, we recorded such a *R. bellii–Legionellaceae* ORF pair. With this approach we identified 15 candidates for HGT between R. bellii and *Legionellaceae*. We performed the same type of analysis for the R. bellii and *Parachlamydiaceae* pair, and found seven candidates for HGT between these bacteria. The list of these 22 HGT-candidate ORFs is given in Table S5. We applied the same protocol to other *Rickettsiales* genomes (Table S6). The numbers of the identified HGT candidates are higher for R. felis (17 ORFs) and Wolbachia pipientis wMel (eight ORFs), and less than six for other *Rickettsiales*. We conducted phylogenetic analyses for all 22 R. bellii HGT candidates. We found nine cases of well-supported clustering (a bootstrap values >80%) of rickettsial sequences exclusively with the sequences of *Legionellaceae* (six cases) or *Parachlamydiaceae* (three cases) (Figure S5). These include rickettsial Sec7 domain–containing proteins (Figure S5H; RBE_0868 for R. bellii), which are homologous to the L. pneumophila RalF protein. RalF is secreted into host cytosol, and used to recruit ADP-ribosylation factor to *Legionella*-containing phagosomes in order to establish a replicative organelle [24]. Cox et al. [25] proposed two events of horizontal transfer for these bacterial Sec7 domain–containing proteins: the first from eukaryotes to bacteria, and the second between *Legionella* and *Rickettsia.* Overall, our results suggest the involvement of HGT for many of the R. bellii genes that are most similar to their homologues in the amoeba-parasites. Most of these HGT events are probably ancient (i.e., before the divergence of *Rickettsia* species), as *Rickettsia* orthologues are always clustered in the phylogenetic trees that we analyzed. We also noted several other groupings of phylogenetically distant organisms (Figure S5A, S5B, S5F, and S5I), including an Entamoeba histolytica protein clustered with bacterial proteins (Figure S5F; 98% bootstrap), suggesting that some of the genes that we analyzed may be prone to lateral transfer.

### R. bellii Replication in Cells

**Figure 7:**
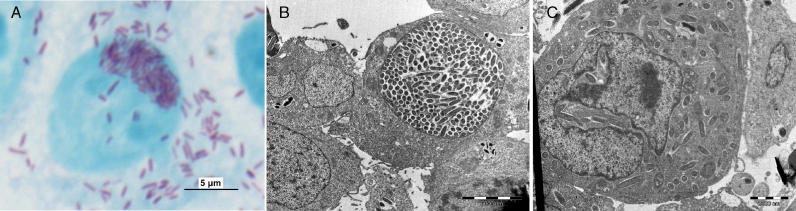
R. bellii Grown in XTC-2 Cell Line (A) Gimenez-stained bacteria observed by optic microscopy. (B) Electronic microscopy of infected cells evidences multiplication of R. bellii within the nucleus. (A) and (B) show the intranuclear accumulation of *R. bellii.* (C) Higher-magnification image showing the intrusion of R. bellii into the nucleus.

R. bellii grows easily in mammalian cells such as Vero cells as well as in *Xenopus* XTC-2 cells. Curiously, we found that R. bellii is very frequently observed in the nuclei (Figure 7). R. bellii appears to reach the nucleus by actin polymerization-based motility. By microscopy, we observed R. bellii forming actin tails (Figure S6). In addition, it exhibits an orthologue (RBE_0855) for R. conorii RickA, which promotes actin polymerization by activating the host Arp2/3 complex [26,27]. After puncturing the nuclear membrane, the bacterium probably becomes trapped inside. Although actin and several actin-related proteins are present in the nucleus, Arp2 and Arp3 are not [28]. R. bellii might thus become trapped within the nucleus due to a reduced actin-based motility caused by the local shortage of Arp2/3 complex. It should be noted that Heinzen et al. reported irregular actin tails of R. rickettsii within the nucleus, but observed no forward movement for the bacteria [29]. Once in the nucleus, R. bellii continues to multiply locally, generating a tight colony within. Progressively, the nucleus inflates as bacteria accumulate, while the size of the cytoplasm of the host cell decreases. Finally, the nuclear membrane is disrupted and the bacteria are released (unpublished data).

In an early characterization of *R. bellii,* Philip et al. observed occasional intranuclear growth of the bacterium [30]. SFG, but not TG, rickettsiae sometimes penetrate and replicate in the nucleus [1]. However, the formation of such an intranuclear colony of bacteria in host cells as observed here for R. bellii has not been previously reported, and appears significantly more frequent for R. bellii than for *R. conorii* on both XTC-2 and Vero cells (Table S7). The concentration of R. bellii in the nucleus at the fifth day after inoculation was ten times larger than that in the cytoplasm of the same cell, demonstrating intranuclear growth (see Materials and Methods, Figure S7, and Table S8). This also suggests that R. bellii divides more rapidly in the nucleus than in the cytoplasm, although it is not ruled out that the apparent faster intranuclear growth of R. bellii could be partially due to its efficient penetration into nuclei compared to R. conorii. Nuclei can be rich in the components of DNA and RNA. It has also been demonstrated that small molecules such as amino acids also accumulate faster in the nucleus than in the cytoplasm of amphibian oocytes [31,32]. R. bellii may be endowed with a specific ability to fully exploit this nutrient-rich environment for its growth.

### Survival of R. bellii in Acanthamoeba polyphaga

We found that *R. bellii* is able to survive for at least 6 wk in *Acanthamoeba polyphaga.* Following the inoculation of 10^6^ amoeba cells with 10^8^ purified *R. bellii, R. bellii* DNA copies were detected by real-time PCR at a stable level for 6 wk. RNA was detected at a stable level for 4 wk and slightly decreased the two following weeks. Subcultures after 2 wk of incubation with amoebae were positive (in XTC-2 cells). In an attempt to cultivate R. bellii in an axenic medium, we previously observed that DNA and RNA levels decreased quickly (unpublished data). Interestingly, we observed R. bellii appearing to form mating pairs by conjugation at the surface of amoebae (Figure S8).

### R. bellii Pathogenicity

We examined the pathogenicity of R. bellii by bacterial inoculation in guinea pigs and rabbits by comparing it to R. conorii (virulent species) and R. montanensis (presumably avirulent species). In both animals, intradermal injection of 50 R. bellii caused slight inflammatory reactions, and inoculation of 50,000 R. bellii induced a black necrotic eschar (Figure S9). This type of eschar is typical of rickettsial pathogenicity and is found in many rickettsioses, including scrub typhus, rickettsialpox, and several tick-borne rickettsioses [1]. Inoculation of at least 5,000 R. conorii induced a black necrotic eschar; inoculation of 50,000 R. montanensis only induced slight inflammatory reactions and no eschar. The histological findings of the cutaneous inoculation area during R. bellii infection were dominated by the presence of inflammatory infiltrates with mainly mononuclear cells (i.e., lymphocytes and macrophages). The epidermis was devoid of necrotic features. Inflammatory infiltrates were seen in the dermis with dermal edema. No vascular injury, vascular thrombosis, or areas of necrosis were observed in the cutaneous tissues. R. bellii was detected in infected cutaneous tissues by immunohistochemical analysis. Numerous rickettsiae were observed in inflammatory cells, mainly macrophages (Figure S9).

The model used herein to test R. bellii pathogenicity was developed specifically for this study. Given that inoculation of rickettsiae occurs through arthropod bites, inoculation by subcutaneous route is representative of the natural infection. Characteristics of the lesions are in favor of an effect due to multiplicating bacteria rather than the sole effect of injected lipopolysaccharide: (1) intact bacteria may be detected after 2 wk by immunohistochemistry in the tissues; (2) an eschar is present (albeit smaller than that observed with R. conorii); and (3) the apparition of the inflammatory lesions and eschar are not immediate. As a pathogenic effect is observed in two different mammal species (i.e., guinea pig and rabbit), R. bellii pathogenesis for humans needs to be further investigated.

## Discussion

R. bellii forms a deep branching lineage within the medically important bacterial genus *Rickettsia.* Sequencing of its genome revealed the first rickettsial *tra* gene cluster likely to encode a complete set of proteins required for conjugal DNA transfer. The *tra* gene cluster recently identified in the pRF plasmid of R. felis [3] is now clearly depicted as a partial form of the *R. bellii tra* gene cluster. Our observation of the sex pili-like appendage supports the functionality of a putative conjugal DNA transfer machinery in *R. bellii.* The lack of genetic transformation tools for rickettsiae has hindered progress in the detailed molecular characterization of the bacteria. The conjugal transfer genes found in R. bellii will provide an additional molecular basis, beside the R. felis plasmid, for the future development of a genetic transformation tool for the study of rickettsiae.

Our phylogenetic analyses suggest lateral transfers of several *tra* genes between the ancestors of rickettsiae and environmental chlamydiae living in amoebae. Previous studies also proposed lateral exchanges for several genes between rickettsiae and chlamydiae, such as the energy uptaking ATP/ADP translocase, a hallmark enzyme of the intracellular parasitism of rickettsiae and chlamydiae. Five paralogous *tlc* genes for ATP/ADP translocase have been found in all sequenced rickettsiae including *R. bellii,* and five paralogues were found in *P. amoebophila.* Phylogenetic analyses suggest that a *tlc* gene has been transferred from the ancestor of chlamydiae to the ancestor of rickettsiae [33,34]. Wolf et al. described two additional clear cases of ancestral gene exchanges between these distantly related bacterial clades [35].

In this study, we observed the high frequency of R. bellii genes exhibiting a strong sequence similarity to the homologues in amoeba-associated bacteria *(L. pneumophila* and *P. amoebophila)* relative to several Alphaproteobacteria outside *Rickettsiales.* A similar but more moderate tendency was observed for many species of *Rickettsiales.* Phylogenetic reconstructions pointed out a possible lateral transfer for many of those genes between the ancestor of *Rickettsia* and the amoeba-parasites. Amoebae are major predators of bacteria in microbial communities, ubiquitous in nature as well as in anthropogenic ecosystems such as tap water and air-conditioning units. However, their significant role as reservoirs and vehicles of (facultative and obligate) intracellular bacteria has been recognized only recently [36,37]. About 20% of the isolates of Acanthamoebae spp. from clinical and environmental sources are found to contain bacterial endosymbionts [37]. Bacteria identified in amoebae cover a wide taxonomic range including Alphaproteobacteria, Betaproteobacteria, Gammaproteobacteria, *Chlamydiales,* and *Bacteroidetes,* although the actual frequency of amoeba-associated bacteria in nature is still unknown [36]. This implies that preferred rickettsial partners for gene exchanges may not be restricted to *Legionellaceae* and *Parachlamydiaceae.* Further studies, including the sequencing of other amoeba-symbionts, are required to better understand the flow of genes between these bacteria, and to obtain clues about shared strategies for intracellular parasitism.

The main compartment for replication within eukaryotic cells is different between *Rickettsia, Legionella,* and *Protochlamydia.* Rickettsiae enter nonphagocytic cells such as intestinal epithelial cells by inducing the formation of phagocytic vacuole and then escape from the membrane-bound vacuole into the host cytoplasm, where they replicate [27]. Members of *Parachlamydiaceae* including P. amoebophila replicate within membrane-bound vacuoles of amoebae [37,38], whereas Legionella pneumophila replicates in vacuoles as well as in the cytoplasm [37]. We speculate that phagocytic vacuoles might have served as an important location for the ancestors of these bacteria to encounter within amoebae. Indeed, in A. polyphaga coinfected by R. bellii and *L. pneumophila,* we observed a co-localization of these bacteria within the vacuole of an amoeba (Figure S10), implying possible interactions of the ancestors of these bacteria in an ancestral amoebal host.

Since many amoeba-associated bacteria or their relatives are pathogens of humans, amoebae have been suggested to act as evolutionary “training grounds” that conferred bacteria the ability to later infect the cells of higher eukaryotes [37,39,40]. Given the recent identification of several uncultured members of *Rickettsiales* within amoebae [41], it is plausible that the ancestors of rickettsiae also used amoebae (or related protozoa) as host, in which they might have encountered other intracellular bacteria. Probably, gene exchanges between these bacteria significantly contributed to their evolution by conferring an immediate selective advantage in the adaptation to the intracellular environment of eukaryotic cells. Such gene exchanges might have been carried out by cross-species conjugation using surface appendages that are similar to those identified today in *R. bellii.*

## Materials and Methods

### Bacterial purification and DNA extraction.

In this study, we used R. bellii RML 369-C strain (Rocky Mountain Laboratory Collection, Golden, Colorado, United States), which was isolated in embryonated chicken eggs from a triturated pool of unfed adult Dermacentor variabilis ticks collected from vegetation near Fayetteville, Arkansas, in June of 1966. R. bellii was cultivated on L929 cells growing on MEM with 4% fetal bovine serum supplemented with 5 mM L-glutamine. The bacteria were treated in the presence of 1% trypsine in K36 buffer for 1 h at 37 °C, then centrifuged and digested by DNAseI for 1 h at 37 °C to reduce eukaryotic DNA contamination. The sample was loaded on two renograffin gradients (26-38-45) and the bands of the purified bacteria were washed in K36, treated again by DNAseI. After inactivation with EDTA 50 mM, the bacteria were resuspended in TE, dispatched in 150 μl tubes, and stored at −80 °C. Depending of this initial concentration, one or two tubes was diluted in 1 ml TNE (10 mM Tris [Ph 7.5], 150 mM NaC1, 2 mM EDTA) and incubated for 5 h at 37 °C in the presence of lysozyme (2 mg/ml). Lysis was performed 2 h at 37 °C by adding 1% SDS and RNAseI (25μg/ml). An overnight treatment with 1mg/ml of proteinase K was followed at 37 °C. After 3 phenol-chloroform extractions and alcohol precipitation, the DNA was resuspended in 30 μl TE, and its concentration was estimated by agarose gel electrophoresis as previously reported [3].

### Shotgun sequencing of R. bellii genome.

Three shotgun genomic libraries were made by mechanical shearing of the DNA using a hydroshear device (GeneMachines, San Carlos, California, United States). Sequence blunt ends were obtained using the T4 DNA polymerase (New England Biolabs, Beverly, Massachusetts, United States) to which the BstXI adaptator was linked. Fragments of 3, 5, and 10 kb were separated on a preparative agarose gel (FMC, Rockland, Maine, United States), extracted with Qiaquick kit (Qiagen, Valencia, California, United States) and ligated into a high copy-number vector pCDNA2.1 (Invitrogen-Life Technologies, Carlsbad, California, United States), digested by BstXI and isolated on another preparative agarose gel. Transformation was performed in the electrocompetent *Escherichia coli* strain *DH10B* (Invitrogen-Life Technologies). Each library was validated on 96 clones after DNA extraction and loading on agarose gel to estimate the real insert size, their homogeneity, and the fraction of empty vector (tolerated up to 5%). Following this quality check of the libraries, we sequenced 96 clones to determine the percentage of eukaryotic DNA contamination from the host cells. Plasmid end-sequencing was carried out using the Big Dye terminator chemistry on an automated ABI 3730 sequencer (PE-Applied Biosystems, Foster City, California, United States). The shotgun libraries of 3, 5, and 10 kb generated 5,416, 5,481, and 5,030 reads, respectively. 16,227 shotgun reads (10× coverage) were analyzed and assembled using Phred, Phrap and Consed software suite [42]. Sequences were retained as valid when a Phred score higher than 20 was associated to at least 75% of the nucleotides. Finishing was performed to improve the low-quality regions, and to fill sequencing and cloning gaps. Specific primers (295) were designed to walk on subcloned DNA and 264 worked successfully. PCR was directly performed on genomic DNA with specific primers and different Taq polymerases depending on the critical region of interest. All finishing sequencing reactions were carried out using the Big Dye chemistry on an automated ABI 3130 sequencer (PE-Applied Biosystems).

The final genome sequence assembly was validated by comparing the restriction patterns obtained by pulsed-field gel electrophoresis and those deduced from the consensus sequence. We analyzed single digests of R. bellii DNA with the following restriction enzymes: ApaI, Afel, FspI, NruI, SbfI, and SgrAI.

### Annotation.

We predicted protein-coding genes (ORFs) using SelfID as previously described [3]. Functional assignments for the ORFs was based on the database searches using BLAST [43] against SWISS-PROT/TrEMBL [44], NCBI/CDD [45], and SMART [46] databases. In most cases, we applied an E-value threshold of 0.001 for the database searches to retrieve homologues. Detailed analyses using multiple sequence alignments and phylogenetic reconstructions were carried out to assign putative functions to the ORFs, when needed. Orthologous gene relationships between R. bellii and other *Rickettsia* species were approximated using the best reciprocal BLAST match criterion. The numbers of transposases, ankyrin/tetratricopeptide repeat–containing genes, and integrases were computed using RPS-BLAST with NCBI/CDD entries related to those domains with a 10^−5^ E-value threshold. tRNA genes were identified using tRNAscan-SE [47]. Repeated DNA sequences were identified using RepeatFinder [48]. To identify *Rickettsia* palindromic elements, we used hidden Markov models [49] based on the previously identified *Rickettsia* palindromic element sequences. ClustalW [50], T-coffee [51], and MUSCLE [52] were used to construct multiple sequence alignments.

### Phylogenetic reconstruction.

For the phylogenetic analyses of the proteins encoded by *tra* genes (Figure S3) and of the proteins recognized as candidates for HGT (Figure S5), we first carried out BLAST searches against the SWISS-PROT/TrEMBL database to retrieve sets of homologues exhibiting high scores to R. bellii ORFs. We used MUSCLE [52] to align the sequences and MEGA [53] to reconstruct neighbor-joining trees with the Jones-Taylor-Thornton (JTT) substitution model. All the gap-containing columns in the alignments were ignored. For the phylogenetic analyses based on the concatenated alignments of multiple proteins (Figures 5 and S4), we chose species based on the results obtained from the analyses of individual proteins; specifically, we included species exhibiting phylogenetic affinities with either R. bellii or P. amoebophila in the trees for the selected six proteins (TraB/C/D/G/H/N). Those species are Sphingomonas aromaticivorans and *Erythrobacter litoralis.* In addition, the analysis of TraN proteins suggests a phylogenetic affinity between P. amoebophila and Azoarcus sp. EbN1. Azoarcus sp. was not included in the multiple-protein analyses due to the lack of the gene for TraD_F_ in this species. Additional analyses using five genes (TraB/C/G/H/N) indicate no specific affinity of Azoarcus sp. EbN1 with R. bellii or P. amoebophila (unpublished data). We conducted phylogenetic analyses with the use of Molphy/ProtML [54] for the maximum likelihood method, FASTME [55] combined with Phylip/Protdist [56] for the balanced minimum evolution method, MEGA for the neighbor-joining method, and the maximum parsimony method. For the distance-based methods and the maximum likelihood method, we used the JTT model of substitution.

### Growth in *Acanthamoeba polyphaga.*

Acanthamoeba polyphaga Linc AP-1 strain was grown in peptone-yeast extract-glucose medium under previously described conditions [57]. A 10^8^ pellet of purified R. bellii was suspended in 10 ml of *Acanthamoeba* starvation medium (Page's amoebal saline [57] with 20 g/l glucose and 2g/l yeast extract). This suspension was then filtered through a 5-μm pore size filter in order to remove remaining cells and put in a 25-cm^2^ cell-culture flask. A pellet containing 10^7^ A. polyphaga previously rinsed twice in Page's amoebal saline was added to this suspension. This test was repeated twice. In the first experiment, the day of inoculation and every 3 d for 2 wk, amoebae were resuspended in the culture medium and 500 μl of suspension were removed. This suspension was titrated by R. bellii quantitative PCR and serially diluted before inoculation into shell-vials with XTC-2 cells (a *Xenopus* cell line) growing at 28 °C. Viability was also tested by RNA detection using RT-PCR targeting 16S rRNA and rompB. The second experiment was performed identically but coculture of R. belli with *Acanthamoeba* was observed for 6 wk and samples for quantitative PCR and RT-PCR were taken every week.

### Detection of actin comet tails generated by *R. bellii.*

Vero cells grown to semiconfluence on glass coverslips were infected with R. bellii for 6 h at 32 °C in humidified CO_2_ incubator (5% CO_2_). Infected cells were then fixed for 1 h at 4 °C with paraformaldehyde (3% w/v in phosphate-buffered saline (PBS) supplemented with 1 mM MgCl_2_ and 1mM CaCl_2_), washed three times with PBS, and then permeabilized with 0.1% Triton X-100 in PBS for 30 s. After three washings in PBS, coverslips were incubated for 45 min at room temperature with a rabbit polyclonal anti-R. bellii Ab (1:1000). Labeling was then revealed with Alexa Fluor 546 goat anti-rabbit IgG (1:300; Invitrogen-Life Technologies) as secondary Ab and actin was stained with FITC-phalloidin (1:250). Both Alexa dye and phalloidin were diluted in DAPI (4′,6′-diamidino-2-phenylindole; Molecular Probes, Eugene, Oregon, United States). After washings, the coverslips were mounted using fluoprep and were examined with a fluorescent microscope using a 100× oil immersion, objective lens.

### Measurement of intranuclear rickettsiae.

We compared the presence of rickettsiae at the fifth day after inoculation using a 10:1 *Rickettsia*–cell ratio. Using transmission electronic microscopy, we found that a higher proportion of XTC-2 cells infected by R. bellii contained bacteria in the nuclei than XTC-2 cells infected by R. conorii (Table S7). These results were confirmed by numerating clusters of Gimenez-stained rickettsiae in both XTC-2 and Vero cells (Table S7). Significantly more R. bellii than R. conorii were found in the nuclei. The pictures of heavily infected XTC-2 cells were analyzed to evaluate the ratio of nuclear to cytoplasmic infections by determining the surface occupied by bacteria in each compartment. Eight and 11 cells infected by R. bellii and R. conorii, respectively, were tested. The mean occupation of the nucleus was 23% for R. bellii and 0.4% for *R. conorii.* In contrast, the cytoplasmic occupation was 1.97 % for R. bellii and 6.7 % for R. conorii (Table S8). This result confirms the intranuclear growth of *R. bellii,* which appears to be faster than the growth within the cytoplasm, and suggests that the intranuclear growth of R. bellii might be more efficient than that of R. conorii in XTC-2 cells.

### Identification of R. bellii pilus by transmission electron microscopy and immunofluorescence assay.

For electron microscopy analysis, supernatant of XTC-2 cells infected for 48 h at 28 °C with R. bellii was carefully collected to minimize the possible shearing of pili. Bacteria were fixed by addition (1:1) of 2% formaldehyde-saline. Samples were applied to copper Formvar grids (Electron Microscopy Sciences, Fort Washington, Pennsylvania, United States) for 2 min and negatively stained with 1% phosphotungstic acid (pH 6.5) for 2–4 min. Samples were examined with a Hitachi 7000 transmission electron microscope (Hitachi, Tokyo, Japan).

Immunofluorescence was performed on 200 μl of similar cultures previously cytocentrifuged on glass slides and fixed by 10 min of incubation with methanol. Staining of bacteria was achieved by incubating slides with a rabbit polyclonal anti–R. belli antibody and with a FITC-conjugated goat anti rabbit Ig diluted in 0.2% Evans blue as secondary antibody. After three washes with PBS, the slides were covered with 5 μM DAPI and observed using an Olympus BX 51 microscope as described above.

### Evaluation of pathogenicity in animals.

All animal experiments were performed in conformity with the laws and regulations controlling experiments with live animals in France and were approved by the French Ministry of Agriculture. The effect of R. bellii inoculation was compared to those of the well-known pathogen R. conorii and to the presumably avirulent *R. montanensis.* Purified suspensions of *R. conorii, R. montanensis* grown in L929 cells, and R. bellii grown in XTC-2 cells titrated at 5 × 10^5^ bacteria/ml were diluted from 10^−1^ to 10^−3^.  Suspensions containing 10^6^ XTC-2 or L929 cells/ml were also prepared and diluted from 10^−1^ to 10^−3^. The backs of male Hartley guinea pigs and of a New Zealand rabbit were shaved. Intradermic inoculations were performed in separate areas with 100 μl of the suspensions of bacteria (5 × 10^4^ to 5 × 10 bacteria inoculated) and with 100 μl of the suspensions of cells used for culture of bacteria and 100 μl of culture medium as controls. All bacteria were inoculated in two guinea pigs. Rabbit was inoculated with R. belli only. Animals were inspected daily to record the aspect of skin at the site of inoculation. A biopsy of eschar was performed at day 5 on guinea pigs and day 10 on rabbit inoculated with R. bellii. At day 14, animals were humanely killed. Areas with eschar or macroscopic inflammation were dissected and put in 10% formalin for histologic study. Formalin-fixed, paraffin-embedded skin biopsy specimens of the cutaneous inoculation area were cut to a thickness of 3 μm and stained with hematoxylin-eosin saffron by use of routine staining methods. Serial sections were also obtained for immunohistochemical investigations. Immunohistochemical analysis was performed by use of a polyclonal mouse antibody directed against R. bellii at a working dilution of 1:500. An immunoperoxidase kit (HistoStain plus kit; Zymed, Montrouge, France) was used in the immunohistological procedure. A negative control was performed using an irrelevant monoclonal mouse antibody.

### Colocalization of R. bellii and Legionella pneumophila in the vacuola of *A. polyphaga.*

A. polyphaga cells were inoculated with R. belli as described above. After 24 h of incubation, 200 μl of a suspension containing 10^6^/ml Legionella pneumophila was added to the amoeba culture flask. After 48 h of additional incubation, infected amoebae were resuspended and 200 μl was used for cytocentrifugation. Slides were fixed for 10 min with methanol. A suspension containing rabbit polyclonal anti–R. belli antibody and mouse polyclonal anti–L. pneumophila antibody diluted 1:200 in PBS with 3% (w/v) nonfat dry milk (100 μl) was added to the slides and incubated in a moist chamber at 37 °C for 30 min. After three washes in PBS, the slides were incubated for 30 min at 37 °C with 100 μl of suspension containing FITC-conjugated goat anti rabbit Ig and Alexa 543–conjugated goat anti mouse Ig diluted 1:100 in PBS containing 0.2% Evans blue. After three washes with PBS, the slides were covered with 5 μM DAPI and the ready-to-use solution “ProLong Gold antifade reagent” (Molecular Probes) and stained for 10 min in the dark.

Cells were observed using an Olympus BX 51 microscope equipped with a 40× UplanFL 1.3 oil lens. Images were acquired using a DS1-QM (Nikon, Melville, New York, United States) cooled (−30 °C) black-and-white camera driven by “Lucia G” software (Nikon and Laboratory Imaging Ltd., Praga, Czech Republic). DAPI-fluorescence images were taken with an excitation cube unit with a single-band–pass (360/55 nm) excitation filter and a band-pass (460/50 nm) barrier filter. Images of FITC–monoclonal antibody were taken with an excitation cube with a single-band–pass (480/20 nm) excitation filter and a band-pass (535/40) barrier filter. Images of Alexa 543 polyclonal antibody were taken with an excitation cube with a single-band–pass (565/30 nm) excitation filter and a high-pass (610 nm) barrier filter. Image analysis was performed using “Lucia G” and ImageJ software (Rasband WS, ImageJ, US National Institutes of Health, Bethesda, Maryland, United States; http://rsb.info.nih.gov/ij). Images were recorded in 12-bit depth with the same exposition parameters.

## Supporting Information

Figure S1 Circular Representation of the R. bellii Genome Circles represent the following from outside to inside: (1) the position along the genome; (2) genes (color-coded according to Cluster of Orthologous Groups categories) along both strands of the genome; (3) tRNAs; (4) rRNA and other RNAs; (5) repeated elements; and (6) GC skew [(G – C) / (G + C)] curve.
(1.1 MB PPT)

Figure S2 Evolutionary Relationships of the Four Transposase Families Found in R. bellii
The phylogenetic trees were generated using the neighbor-joining method and the JTT substitution matrix. Proteins homologous to the R. bellii transposases were searched in other bacteria by screening the Genpept database using BLAST. Multiple alignments were carried out using the MUSCLE program. (A), (B), (C), and (D) correspond to families 1, 2, 3, and 4 in the text. Some rickettsial fragmented transposases were not included in the trees.
(80 KB PPT)

Figure S3 Phylogenetic Trees of the Individual Proteins Encoded in the *tra* Gene Clusters (A) TraG_F_, (B) TraC_F_, (C) TraN_F_, (D) TraD_F_, (E) TraB_F_, (F) TraH_F_, (G) TraU_F_, (H) TraF_F_, (I) TraW_F_, (J) TraE_F_, (K) TrbC_F_, (L) TraA_Ti_, (M) TraD_Ti_. The tree was generated using the neighbor-joining method with the JTT model and midpoint rooting.
(149 KB PPT)

Figure S4 Phylogenetic Tree of the Proteins Encoded in the F-Like *tra* Gene Clusters The tree was built using a maximum likelihood method with JTT model and midpoint rooting from the concatenated sequence alignment of TraB_F_, TraC_F_, TraD_F_, TraG_F_, TraH_F_, and TraN_F_. Plasmid-encoded sequences are indicated by asterisks. Other sequences are encoded in chromosomes.
(34 KB PPT)

Figure S5 Phylogenetic Trees of the HGT Candidate Proteins (A) RBE_0090, (B) RBE_0387, (C) RBE_0461, (D) RBE_0462, (E) RBE_0486, (F) RBE_0692, (G) RBE_0860, (H) RBE_0868, (I) RBE_1344. The trees were generated using the neighbor-joining method with the JTT model and midpoint rooting.
(153 KB PPT)

Figure S6 Actin Comet Tails of R. bellii
Vero cells infected for 6 h with R. bellii showing actin (phaloidine-FITC staining, in green) and bacteria (polyclonal anti–R. bellii revealed with Alexa Fluor 546 goat anti-rabbit IgG, in red). Nucleic acids were stained with DAPI (blue). White arrowheads point to actin tails.
(1.6 MB PPT)

Figure S7 Detection of R. belli in Vero Cell Nucleus Bacteria were detected within Vero cells infected for 5 d with R. bellii and gently permeabilized with Triton before incubation with a rabbit polyclonal anti–R. bellii Ab (1:1,000) revealed by an Alexa 546–coupled anti-rabbit (1:300) as secondary Ab. Magnification 100×.
(896 KB PPT)

Figure S8 Immunofluorescence Detection Using Coupled FITC-Specific Polyclonal Antibodies of R. bellii (Stained Green) Shows Two R. bellii Cells Apparently in a Conjugation Process at the Surface of an *Acanthamoeba*
Amoeba nucleotides (mostly in the nucleus) are stained blue by DAPI, and L. pneumophila are stained red (immunofluorescence detection with Alexa 543 coupled–specific polyclonal antibodies).
(1.3 MB PPT)

Figure S9 Pathogenicity of R. bellii on a Guinea Pig (A) Inoculation eschars on a guinea pig injected intradermally with increasing dilutions of R. bellii (small central eschar) and R. conorii (large eschar) suspensions*.* The same dilutions of R. montanensis induced a slight inflammation only with the highest dose. This picture was taken 6 d after inoculation. The numbers of inoculated bacteria are indicated. (B) Immunohistochemistry of the escarotic lesion using anti–R. bellii antibodies.
(8.0 MB PDF)

Figure S10 Colocalization of R. bellii and L. pneumophila in A. polyphaga
Immunofluorescence detection using specific rabbit polyclonal antibodies against R. bellii (FITC-coupled antibodies, stained green) and specific mouse polyclonal antibodies against L. pneumophila (Alexa 543–coupled antibodies, stained red) shows colocalization within a vacuole of *A. polyphaga.* Amoeba nucleotides (mostly in the nucleus) are stained blue by DAPI.
(1.8 MB PPT)

Table S1 Tick Hosts of R. bellii
(39 KB DOC)

Table S2 R. bellii–Specific Genes with Putative Functional Assignments (22 KB DOC)

Table S3 Distribution of *sca* Genes among *Rickettsia* Genomes (45 KB DOC)

Table S4 Bootstrap Supports for the Clustering of *Rickettsia* and P. amoebophila by Different Tree Inference Methods Applied to the Concatenated Sequence Alignments (23 KB DOC)

Table S5 Candidate R. bellii ORFs for HGT (25 KB DOC)

Table S6 Number of HGT Candidates Identified by Reciprocal BLAST Searches (25 KB DOC)

Table S7 Statistical Analysis of the Association of Rickettsiae with the Nucleus of Infected Cells (27 KB DOC)

Table S8 Nucleus versus Cytoplasm Surface Occupation by Rickettsia (26 KB DOC)

### Accession Number

The GenBank (http://www.ncbi.nlm.nih.gov/Genbank) accession number for the R. bellii genome is CP000087.

## Abbreviations

HGT: horizontal gene transfer
JTT: Jones-Taylor-Thornton
ORF: open reading frame
PBS: phosphate-buffered saline
SFG: spotted fever group
T4SS: type IV secretion system
TA: toxin antitoxin
TG: typhus group
