# Supplementary material for: Genome Sequence of Rickettsia bellii Illuminates the Role of Amoebae in Gene Exchanges between Intracellular Pathogens
Source: PLoS Genet. 2006 May 12;2(5):e76. doi: 10.1371/journal.pgen.0020076 (PMC1458961; doi:10.1371/journal.pgen.0020076)
Supplement: Supplementary file 1 [file pgen.0020076.sg009.pdf]

(A)

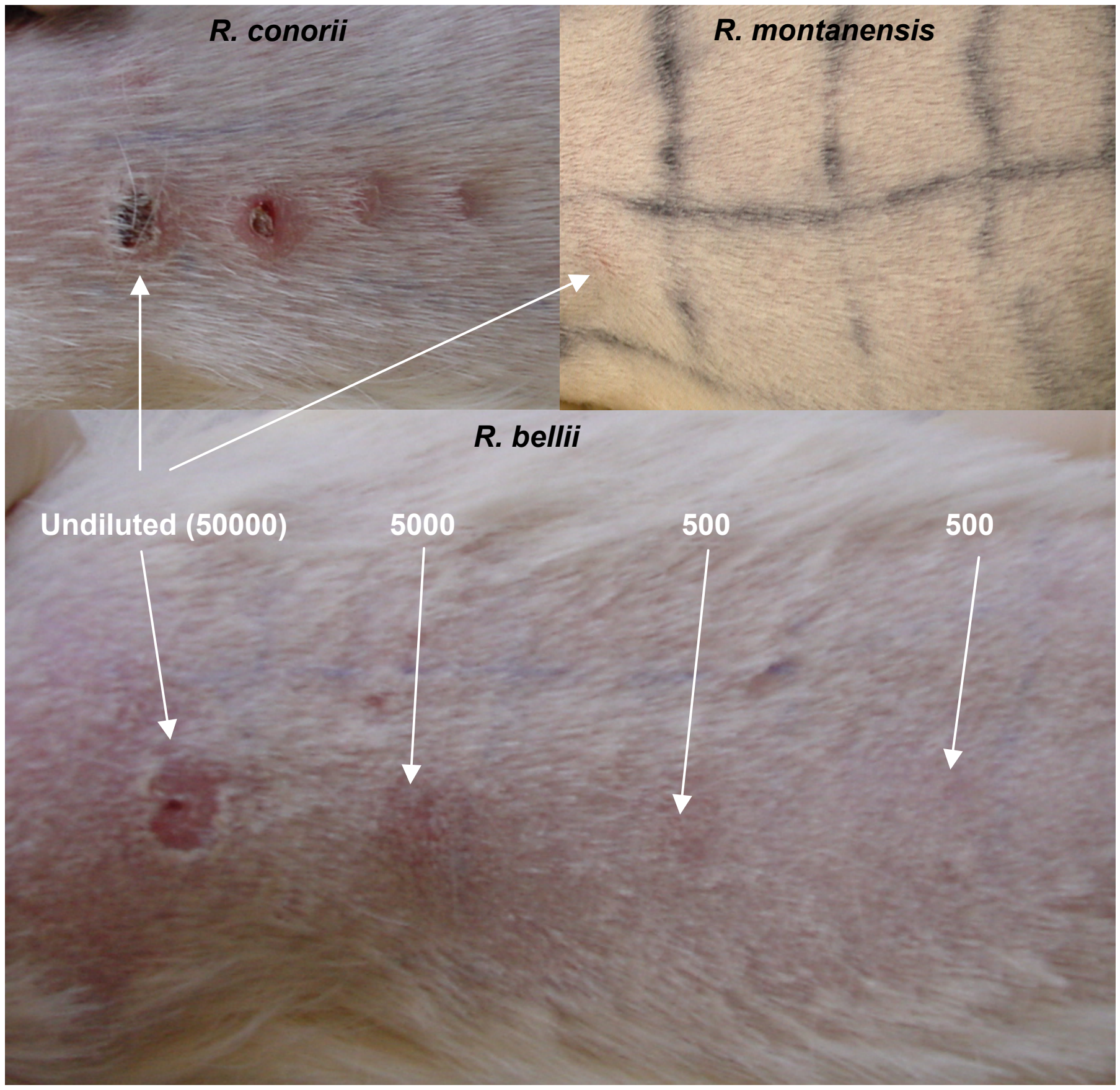
R. conorii
R. montanensis
R. bellii
Undiluted (50000)
5000
500
500

(B)

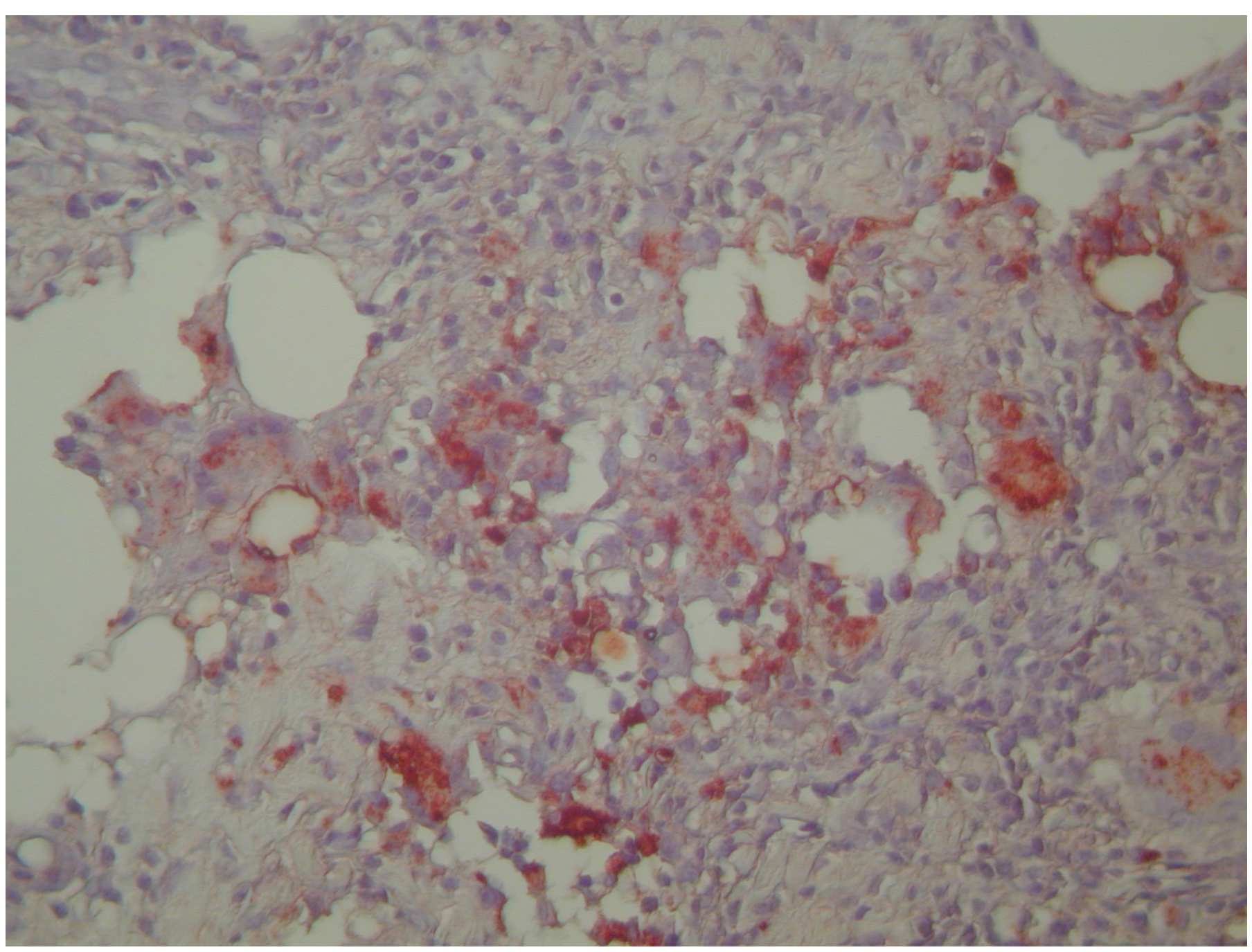

Fig. S9
