# Supplementary material for: Genome Sequence of Rickettsia bellii Illuminates the Role of Amoebae in Gene Exchanges between Intracellular Pathogens
Source: PLoS Genet. 2006 May 12;2(5):e76. doi: 10.1371/journal.pgen.0020076 (PMC1458961; doi:10.1371/journal.pgen.0020076)
Supplement: Supplementary file 2 [file pgen.0020076.st001.doc]

**Table S1.** Tick hosts of *R. bellii*.

| Tick | **Place** | **References** |
| --- | --- | --- |
| **Ixodid ticks (hard ticks)** |  |  |
| *Dermacentor andersonii* | USA | Ref. S1 |
| *D. variabilis* | USA | Ref. S1 |
| *D. occidentalis* | USA | Ref. S1 |
| *D. parumapertus* | USA | Ref. S1 |
| *D. albipictus* | USA | Ref. S1 |
| *Amblyomma* species | Brazil | Ref. S2,3 |
| *Hemaphysallis* species | USA | [30] |
| **Argasid ticks (soft ticks)** |  |  |
| *Argas cooleyi* | USA | [30] |
| *Ornithodoros concanensis* | USA | [30] |

**References:**

S1. Philip RN, Casper EA (1981) Serotypes of spotted fever group rickettsiae isolated from Dermacentor andersoni (Stiles) ticks in western Montana. Am J Trop Med Hyg 30: 230–238.

S2. Labruna MB, Whitworth T, Bouyer DH, McBride J, Camargo LM, et al. (2004) Rickettsia bellii and Rickettsia amblyommii in Amblyomma ticks from the State of Rondonia, Western Amazon, Brazil. J Med Entomol 41: 1073–1081.

S3. Labruna MB, McBride JW, Bouyer DH, Camargo LM, Camargo EP, et al. (2004) Molecular evidence for a spotted fever group Rickettsia species in the tick Amblyomma longirostre in Brazil. J Med Entomol 41: 533–537.
