# Supplementary material for: Genome Sequence of Rickettsia bellii Illuminates the Role of Amoebae in Gene Exchanges between Intracellular Pathogens
Source: PLoS Genet. 2006 May 12;2(5):e76. doi: 10.1371/journal.pgen.0020076 (PMC1458961; doi:10.1371/journal.pgen.0020076)
Supplement: Supplementary file 3 [file pgen.0020076.st002.doc]

## Table S2. *R. bellii* specific genes with putative functional assignments.

| ORF ID | Predicted function |
| --- | --- |
| RBE_0007 | Acyltransferase family protein |
| RBE_0106 | Deoxyribodipyrimidine photo-lyase PhrB (EC 4.1.99.3) |
| RBE_0152 | SMR-type multi-drug efflux transporter |
| RBE_0153 | Phosphoglycerol transferase and related proteins |
| RBE_0252 | Putative transcriptional regulator |
| RBE_0254 | Predicted esterase of the alpha/beta hydrolase fold |
| RBE_0269 | Phospholipase/lecithinase/hemolysin |
| RBE_0380 | Putative transcriptional regulator |
| RBE_0763 | ABC-type anion transport system permease component |
| RBE_0764 | ABC-type anion transport system permease component |
| RBE_0883 | Cytosine-C5 specific DNA methylase |
| RBE_0892 | RDD family protein |
| RBE_1344 | EMAP domain containing protein |
| RBE_1400 | Putative hemagglutinin protein |
| RBE_1408 | Phenylalanine-4-hydroxylase (EC 1.14.16.1) |
