# Supplementary material for: Genome Sequence of Rickettsia bellii Illuminates the Role of Amoebae in Gene Exchanges between Intracellular Pathogens
Source: PLoS Genet. 2006 May 12;2(5):e76. doi: 10.1371/journal.pgen.0020076 (PMC1458961; doi:10.1371/journal.pgen.0020076)
Supplement: Supplementary file 4 [file pgen.0020076.st003.doc]

**Table S3.** Distribution of *sca* genes among *Rickettsia* genomes.

| ***sca* gene** | **RFE** | **RCO** | **RPR** | **RTY** | **RBE** | **RBE ORF ID** | **RBE ORF size (aa)** | **Longest ORF size (aa) ^a^** |
| --- | --- | --- | --- | --- | --- | --- | --- | --- |
| *sca0* (*ompA*) | S | C | - | - | - | - |  | 2,021 (RCO) |
| *sca1* | C | C | S | C | S | RBE_0170 | 594 | 1,902 (RCO) |
| *sca2* | C | C | - | - | C | RBE_1280 *^b^* | 909 *^b^* | 1,795 (RCO) |
| *sca3* | C | S | C | C | S | RBE_0761/0762 | 253/488 | 3,122 (RFE) |
| *sca4* (gene D) | C | C | C *^c^* | C | C | RBE_0769 | 1,122 | 1,122 (RBE) |
| *sca5* (*ompB*) | C | C | C | C | C | RBE_0184 | 913 | 1,655 (RCO) |
| *sca6* | - | - | S | C | C | RBE_1280 *^b^* | 909 *^b^* | 1,483 (RTY) |
| *sca7* | S | - | - | - | - | - |  | 103-880 (RFE) |
| *sca8* | C | S | - | - | S | RBE_0355/0354/0353 | 669/53/143 | 1,502 (RFE) |
| *sca9* | C | S | - | - | S | RBE_0146/0147 | 354/408 | 557 (RFE) |
| *sca10* | S | S | - | - | - | - |  | 107-492 (RFE) |
| *sca11* | C | - | - | - | - | - |  | 333 (RFE) |
| *sca12* | C | - | - | - | - | - |  | 476 (RFE) |
| *sca13* | C | S | - | - | S | RBE_0148/0149 | 640/1981 | 2,199 (RFE) |
| *sca14* | - | - | - | - | C | RBE_0384 | 366 | 366 (RBE) |
| *sca15* | - | - | - | - | C | RBE_0918 | 630 | 630 (RBE) |
| *sca16* | - | - | - | - | C | RBE_0270 | 186 | 186 (RBE) |

Abbreviations are as follows. Rfe: *R. felis*; Rco: *R. conorii*; Rpr: *R. prowazekii*; Rty: *R. typhi*; Rbe: *R. bellii*; C: complete gene; S: split or fragmented gene; ‘-‘: absent.

*^a^* Species exhibiting the longest ORF is indicated between parentheses.

*^b^* *R. bellii* exhibits a pre-duplication form *sca2* and *sca6*.

*^c^* According to our recent re-sequencing (GenBank: AY964638).
