# Supplementary material for: Genome Sequence of Rickettsia bellii Illuminates the Role of Amoebae in Gene Exchanges between Intracellular Pathogens
Source: PLoS Genet. 2006 May 12;2(5):e76. doi: 10.1371/journal.pgen.0020076 (PMC1458961; doi:10.1371/journal.pgen.0020076)
Supplement: Supplementary file 5 [file pgen.0020076.st004.doc]

**Table S4.** Bootstrap supports for the clustering of *Rickettsia* and *P. amoebophila* by different tree inference methods applied to the concatenated sequence alignments.

| **Clade** | **Data set** | **ML** | **BME** | **NJ** | **MP** |
| --- | --- | --- | --- | --- | --- |
| (*R. bellii*, *P. amoebophila*) | Six proteins (TraG_F_/C_F_/N_F_/D_F_/B_F_/H_F_) | 100% | 100% | 100% | 93% |
| (*Rickettsia*, *P. amoebophila*) | Two proteins (TraG_F_/D_F_) | 95% | 100% | 97% | 41% |
| (*R. bellii*, *R. felis*) | Two proteins (TraG_F_/D_F_) | 100% | 100% | 100% | 100% |

ML: Maximum Likelihood method (ProtML); BME: Balanced Minimum Evolution method (FastME); NJ: Neighbor Joining method (MEGA); MP: Maximum Parsimony method (MEGA).
