# Supplementary material for: Genome Sequence of Rickettsia bellii Illuminates the Role of Amoebae in Gene Exchanges between Intracellular Pathogens
Source: PLoS Genet. 2006 May 12;2(5):e76. doi: 10.1371/journal.pgen.0020076 (PMC1458961; doi:10.1371/journal.pgen.0020076)
Supplement: Supplementary file 6 [file pgen.0020076.st005.doc]

**Table S5.** Candidate *R. bellii* ORFs for horizontal gene transfer.

| ***R. bellii*** | Annotation | **BLAST best hit species** | **SWISS-PROT, TrEMBL ID** | **Annotation** |
| --- | --- | --- | --- | --- |
| RBE_0090 | Strees induced DNA-binding protein (Dps family) | *Legionella pneumophila* subsp. *pneumophila* (strain Philadelphia 1) | Q5ZXP2_LEGPH | DNA binding stress protein. |
| RBE_0107 | blaD; Beta-lactamase (Class D) | *Legionella pneumophila* (strain Lens) | Q5WWP7_LEGPL | Hypothetical protein. |
| RBE_0387 | ABC transporter ATP-binding protein | *Protochlamydia amoebophila* UWE25 | Q6MCG4_PARUW | Hypothetical protein. |
| RBE_0432 | traC; Conjugative transfer protein TraC | *Protochlamydia amoebophila* UWE25 | Q6MB95_PARUW | Putative inner-membrane protein traC. |
| RBE_0446 | Unknown | *Legionella pneumophila* subsp. *pneumophila* (strain Philadelphia 1) | Q5ZS41_LEGPH | Hypothetical protein. |
| RBE_0460 | Unknown | *Protochlamydia amoebophila* UWE25 | Q6MA63_PARUW | Hypothetical protein. |
| RBE_0461 | Acetyltransferase | *Legionella pneumophila* subsp. *pneumophila* (strain Philadelphia 1) | Q5ZTJ0_LEGPH | Hypothetical protein. |
| RBE_0462 | Unknown | *Protochlamydia amoebophila* UWE25 | Q6MBI9_PARUW | Putative aminoglycoside N6'-acetyltransferase. |
| RBE_0486 | Bacterial transcription activator, effector binding domain | *Protochlamydia amoebophila* UWE25 | Q6MF60_PARUW | Hypothetical protein. |
| RBE_0488 | mazG; MazG-like (predicted pyrophosphatase) domain | *Legionella pneumophila* subsp. *pneumophila* (strain Philadelphia 1) | Q5ZTU3_LEGPH | MazG (Nucleoside triphosphate pyrophosphohydrolase). |
| RBE_0521 | abcT3; Multidrug resistance ABC transporter ATP-binding protein | *Legionella pneumophila* (strain Paris) | Q5WS79_LEGPA | Hypothetical protein. |
| RBE_0630 | Unknown | *Legionella pneumophila* (strain Paris) | Q5X7H3_LEGPA | Hypothetical protein. |
| RBE_0651 | Glycosyltransferase | *Legionella pneumophila* (strain Lens) | Q5WSR1_LEGPL | Hypothetical protein. |
| RBE_0692 | Unknown | *Legionella pneumophila* (strain Paris) | Q5WS38_LEGPA | Hypothetical protein. |
| RBE_0860 | Acetyltransferase | *Legionella pneumophila* (strain Paris) | Q5WS26_LEGPA | Hypothetical protein. |
| RBE_0868 | Sec7 domain containing protein | *Legionella pneumophila* subsp. *pneumophila* (strain Philadelphia 1) | Q5ZU58_LEGPH | Guanine nucleotide exchange protein. |
| RBE_0942 | Unknown | *Protochlamydia amoebophila* UWE25 | Q6MBB2_PARUW | Hypothetical protein. |
| RBE_1079 | Predicted transcriptional regulator | *Protochlamydia amoebophila* UWE25 | Q6MCI0_PARUW | Hypothetical protein. |
| RBE_1189 | lpxD; UDP-3-O-[3-hydroxymyristoyl] glucosamine N-acyltransferase [EC:2.3.1.-] | *Legionella pneumophila* subsp. *pneumophila* (strain Philadelphia 1) | Q5ZRD8_LEGPH | UDP-3-O-[3-hydroxymyristoyl] glucosamine N-acyltransferase (EC 2.3.1.-). |
| RBE_1194 | rssA; Predicted esterase of the alpha-beta hydrolase superfamily | *Legionella pneumophila* (strain Lens) | Q5WX63_LEGPL | Hypothetical protein. |
| RBE_1344 | EMAP domain containing protein (CsaA chaperon-like protein) | *Legionella pneumophila* (strain Lens) | Q5WTQ5_LEGPL | Hypothetical protein. |
| RBE_1423 | Unknown | *Legionella pneumophila* (strain Paris) | Q5X5B4_LEGPA | Hypothetical protein. |
