# Supplementary material for: Genome Sequence of Rickettsia bellii Illuminates the Role of Amoebae in Gene Exchanges between Intracellular Pathogens
Source: PLoS Genet. 2006 May 12;2(5):e76. doi: 10.1371/journal.pgen.0020076 (PMC1458961; doi:10.1371/journal.pgen.0020076)
Supplement: Supplementary file 7 [file pgen.0020076.st006.doc]

**Table S6.** Number of HGT candidates identified by reciprocal BLAST searches.

|  | RBE | **RFE** | **RCO** | **RPR** | **AMA** | **ECA** | **ERA** | **ERG** | **ERF** | **WBM** | **WME** |
| --- | --- | --- | --- | --- | --- | --- | --- | --- | --- | --- | --- |
| *Legionellaceae* (Family) | 15 | 16 | 5 | 5 | 0 | 0 | 0 | 0 | 0 | 0 | 5 |
| *Parachlamydiaceae* (Family) | 7 | 1 | 0 | 0 | 0 | 1 | 1 | 1 | 1 | 2 | 3 |

RBE: *Rickettsia bellii*; RFE: *Rickettsia felis*; RCO: *Rickettsia conorii*; RPR: *Rickettsia prowazekii*; AMA: *Anaplasma marginale*; ECA: *Ehrlichia canis*; ERA: *Ehrlichia ruminantium* Wolgevonden (South Africa); ERG: *Ehrlichia ruminantium* Gardel; ERF: *Ehrlichia ruminatium* Welgevonden (France); WBM: *Wolbachia* wBm; WME: *Wolbachia* wMel
