# Supplementary material for: Genome Sequence of Rickettsia bellii Illuminates the Role of Amoebae in Gene Exchanges between Intracellular Pathogens
Source: PLoS Genet. 2006 May 12;2(5):e76. doi: 10.1371/journal.pgen.0020076 (PMC1458961; doi:10.1371/journal.pgen.0020076)
Supplement: Supplementary file 8 [file pgen.0020076.st007.doc]

**Table S7.** Statistical analysis of the association of rickettsiae with the nucleus of infected cells.

| **Counting method** | **Cell line** | **Number of tested cells**  **(*R. belii / R. conorii*)** | **Rickettsiae in the nucleus of infected cells** | | ***P-* value** |
| --- | --- | --- | --- | --- | --- |
|  |  |  | ***R. bellii*** | ***R. conorii*** |  |
| **Electron microscopy** |  |  |  |  |  |
| Any rickettsia in the nucleus | XTC2 | 137 / 116 | 34 (25 %) | 16 (14 %) | 0.04 |
| More than 6 rickettsiae in the nucleus | XTC2 | 137 / 116 | 19 (14 %) | 2 (1.7 %) | < 10^-2^ |
| **Optical microscopy**  (Gimenez stain) |  |  |  |  |  |
| Clusters of > 10 rickettsiae | XTC2 | 314 / 300 | 25 (7 %) | 0 (0 %) | < 10^-2^ |
| Clusters of > 10 rickettsiae | Vero | 200 / 300 | 48 (19 %) | 2 (0.7 %) | < 10^-2^ |
