# Supplementary material for: Genome Sequence of Rickettsia bellii Illuminates the Role of Amoebae in Gene Exchanges between Intracellular Pathogens
Source: PLoS Genet. 2006 May 12;2(5):e76. doi: 10.1371/journal.pgen.0020076 (PMC1458961; doi:10.1371/journal.pgen.0020076)
Supplement: Supplementary file 9 [file pgen.0020076.st008.doc]

**Table S8.** Nucleus *vs.* cytoplasm surface occupation by rickettsia.

|  | **Number of XTC2 cells tested** |  | **Total surfaces** | | | **Surfaces occupied by rickettsiae** | | **Ratio of Rickettsia/total surface** | |
| --- | --- | --- | --- | --- | --- | --- | --- | --- | --- |
|  |  |  | **Cell** | **Cytoplasm** | **Nucleus** | **Nucleus** | **Cytoplasm** | **Nucleus** | **Cytoplasm** |
| *R. bellii* | 8 | Mean | 86 | 51 | 35 | 10 | 1 | 23 % | 2 % |
|  |  | Standard deviation | 46 | 24 | 24 | 14 | 1 | 16 % | 3 % |
| *R. conorii* | 11 | Mean | 123 | 88 | 35 | 0.1 | 6 | 0.4 % | 7 % |
|  |  | Standard deviation | 37 | 33 | 12 | 0.2 | 4 | 0.5 % | 4 % |

An arbitrary unit is used for surface area.
